# *Helicobacter pylori* infection: is there circulating vacuolating cytotoxin A or cytotoxin-associated gene A protein?

**DOI:** 10.1186/s13099-022-00519-8

**Published:** 2022-12-03

**Authors:** Ichiro Imoto, Satoko Oka, Masaki Katsurahara, Misaki Nakamura, Taro Yasuma, Junko Akada, Corina N. D’Alessandro-Gabazza, Masaaki Toda, Noriyuki Horiki, Esteban C. Gabazza, Yoshio Yamaoka

**Affiliations:** 1 Digestive Endoscopy Center, Department of Internal Medicine, Doshinkai Tohyama Hospital, Minamishinmachi 17-22, Tsu, Mie 514-0043 Japan; 2 Department of Immunology, Mie University Faculty and Graduate School of Medicine, Mie University Hospital, Edobashi 2-174, Tsu, Mie 514-8507 Japan; 3 Department of Gastroenterology and Hepatology, Mie University Faculty and Graduate School of Medicine, Mie University Hospital, Edobashi 2-174, Tsu, Mie 514-8507 Japan; 4 Department of Environmental and Preventive Medicine, Oita University Faculty of Medicine, 1-1 Idaigaoka, Hasama-Machi, Yufu, Oita 879-5593 Japan; 5 Department of Medicine, Gastroenterology and Hepatology Section, Baylor College of Medicine, Houston, TX USA

**Keywords:** Vacuolating cytotoxin A (VacA), Cytotoxin-associated gene A (CagA), *Helicobacter pylori*, Cytokine, Systemic circulation, Extragastric disease

## Abstract

**Background:**

*Helicobacter pylori* infection is a well-recognized cause of gastric diseases, including chronic gastritis, peptic ulcer, and gastric cancer. Vacuolating cytotoxin-A (VacA) and cytotoxin-associated gene A protein (CagA) play a role in the pathogenesis of *H. pylori*-related gastric diseases. Also, extragastric disorders are frequent morbid complications in patients with *H. pylori* infection. However, the direct pathologic implication of these virulence factors in extragastric manifestations remains unclear. Our hypothesis in the present study is that VacA and CagA released by *H. pylori* in the gastric mucosa leak into the systemic circulation, and therefore they can be measured in serum.

**Results:**

Sixty-two subjects were enrolled. They were allocated into *the H. pylori-positive and H. pylori-negative groups*. VacA and CagA were measured by immunoassays. The serum levels of VacA and CagA above an upper limit cut-off (mean plus two standard deviations of the mean in patients without *H. pylori* infection) were considered positive for antigen circulating level. Five out of 25 *H. pylori*-positive patients were positive for both serum VacA and serum CagA. The serum levels of VacA and CagA were significantly correlated with the serum levels of anti- *H. pylori* antibody and interleukin-12p70 among all *H. pylori-positive and H. pylori*-negative patients.

**Conclusions:**

This study suggests that spill-over of VacA and CagA antigens in the systemic circulation may occur in some patients with *H. pylori* infection.

## Introduction

*Helicobacter pylori* (*H. pylori*) infection is the leading cause of gastroduodenal diseases [1–4]. Mendall et al. first reported the association between *H. pylori* infection and extra-gastric diseases in 1994 [5]. Several studies have demonstrated the role of *H. pylori* in extra-gastric diseases, including hematological, cardiovascular, neurologic, dermatologic, immunologic, ocular, and metabolic disorders [5–13]. In 1988, Leunk et al. reported that supernatants from *H. pylori* broth culture contain a proteinaceous component and named it "vacuolating cytotoxin" (VacA) because it causes vacuolization of cultured eukaryotic cells [14]. Another virulence factor, the effector protein cytotoxin-associated gene A (CagA) encoded by the *cag* pathogenic island (cag PAI), which is a type IV secretion system, is linked to a higher risk of gastric cancer or peptic ulcer disease [15, 16]. Now, we know that both VacA and CagA are the major pathogenic factors involved in *H. pylori*-related diseases [16, 17]. Increasing evidence also implicates *H. pylori*-derived proteins in the enhanced concentration of inflammatory mediators, platelet stimulation, and coagulation system activation in systemic circulation [18]. However, whether the systemic and extragastric manifestations associated with *H. pylori* infection are caused by *H. pylori* virulence factors expressed locally at gastric mucosa or by the spillover of *H. pylori* virulence factors into the circulation is unknown. To gain some insight, in the present study, we developed immunoassays to measure the levels of VacA and CagA antigens in systemic circulation.

## Materials and methods

### Subjects

This study included 62 subjects (males 38, females 24) that consulted our hospital from November 2016 through April 2017. All subjects underwent blood sampling and esophagogastroduodenoscopy. There were 40 outpatients, 1 inpatient, and 21 periodical health checkup participants among all subjects. Subjects that underwent a routine health check-up were included in the study because the number of patients negative for *H. pylori* was very low. The mean age of all subjects was 55.98 ± 1.71 years old. The exclusion criteria were treatment with a proton pump inhibitor, potassium competitive acid blocker, or antibiotics within the last month. Thirteen subjects received *H. pylori* eradication therapy. Of these, 11 subjects had successful eradication and two unsuccessful eradication therapy.

### Esophagogastroduodenoscopy

Two expert physicians performed the esophagogastroduodenoscopy using the Olympus Evis Lucera Elite system with a GIF-H290 endoscope (Olympus Corporation, Tokyo, Japan). This endoscopic study was performed in all subjects that provided informed consent and after obtaining permission from the institutional Ethics Committee. Subjects fasted from 21:00 of the previous day until the time of the endoscopic procedure. Before the procedure, subjects received pharyngeal anesthesia with lidocaine hydrochloride. We checked endoscopic findings and performed urea breath tests as needed to evaluate *H. pylori* infection status. Briefly, endoscopic findings in uninfected individuals reveal a regular arrangement of collecting venules (RAC) on the gastric angle and a closed type (C_0_-C_1_) atrophic pattern according to the Kimura-Takemoto classification [19, 20]. On the other hand, endoscopic findings in *H. pylori*-infected subjects reveal extensive gastric atrophy on the corpus and cardia. Endoscopy is a reliable method with high reproducibility that can accurately predict the presence of histological atrophy [21–23]. Endoscopy is a common procedure during health check-ups in Japan due to the high frequency of gastric cancer [24].

### Analysis of serum anti-*H. pylori* antibody and patient group allocation

**Fig. 1:**
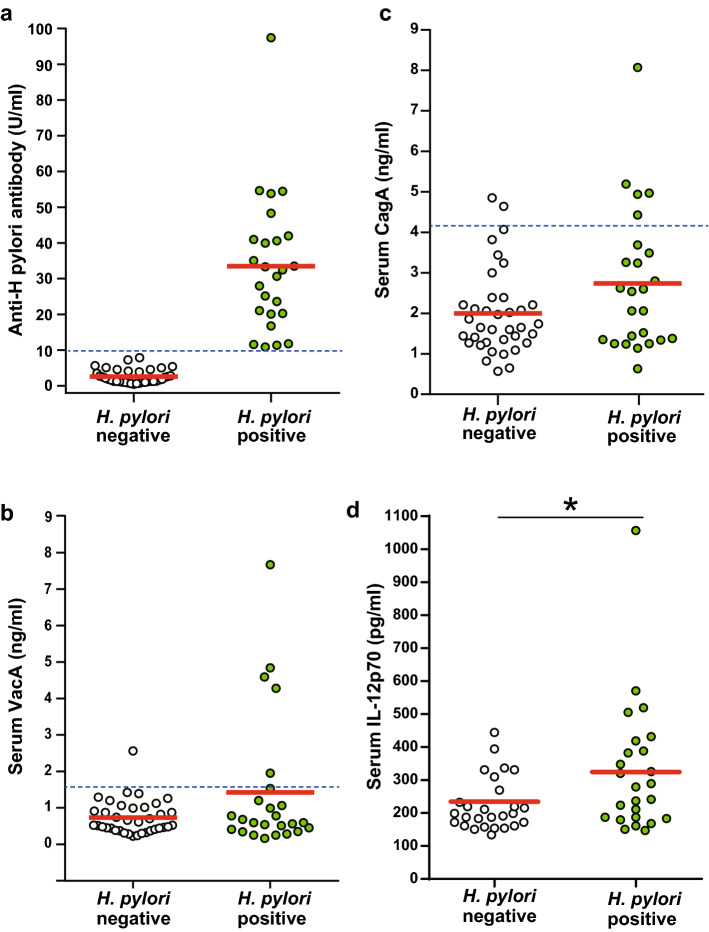
Serum concentration of anti-*Helicobacter pylori* antibody, vacuolating cytotoxin A, cytotoxin-associated gene A protein and interleukin-12p70. Anti-*Helicobacter pylori* antibody (**a**), vacuolating cytotoxin A (VacA) (**b**), cytotoxin-associated gene A protein (CagA) (**c)** and interleukin-12p70 (IL-12) (**d)** were measured using immunoassays. Solid red lines indicate the means, and blue dotted lines indicate upper cutoff values. Statistical analysis by two-tailed unpaired t-test. *p < 0.05

Blood was sampled and collected in vacutainers without anticoagulants. After centrifugation (400 xg, 4 °C, 10 min), serum was separated and stored at − 20 °C until use. We measured the serum anti-*H. pylori* antibody titer with an enzyme-linked immunoassay kit using antigens derived from Japanese individuals (E-plate Eiken *H. pylori* antibody II kit, Eiken Chemical, Tokyo, Japan). Most specialists in Japan used this kit to measure the serum antibody titer. The assay has a range of ≥ 3 U/mL–< 100 U/ml. The manufacturer recommends a cut-off value of 10 U/mL to diagnose *H. pylori* positivity [25]. Therefore, in the present study, we allocated the subjects into two groups: an *H. pylori*-positive group with anti-*H. pylori* antibody of ≥ 10 U/ml, and an *H. pylori*-negative group with anti-*H. pylori* antibody of ≤ 9.9U/mL (Fig. 1a). However, it is worth noting here that studies conducted using this kit showed that *H. pylori*-positive patients with atrophic gastritis, intestinal metaplasia, or gastric cancer might show serum titers in the range of 3–9.9 U/mL [24].

### Analysis of circulating VacA and CagA

The serum concentrations of VacA and CagA were measured by immunoassays using anti-VacA and anti-CagA antibodies as capture antibodies (AUSTRAL Biologicals, San Ramon, CA, USA) and their biotinylated forms as detecting antibodies. The serum concentration of each virulence factor was extrapolated from a standard curve prepared using commercially available antigens (AUSTRAL Biologicals (San Ramon, CA, USA). Concentrations of VacA (0.729 + 0.468 × 2 = 1.665 ng/ml) and CagA (1.998 + 1.057 × 2 = 4.112 ng/ml) antigens above an upper limit cut-off (the mean + two standard deviations of the mean in subjects without *H. pylori* infection) were considered positive for circulating antigen level. Inter-assay and intra-assay variabilities were less than 10%. The serum concentration of interleukin (IL)-12 was measured using a commercially available immunoassay kit (BD Biosciences, San Jose, CA, USA).

### Statistical analysis

Data are expressed as the mean ± the standard errors of the mean (S.E.M.). The sample size was calculated using the G*Power 3.1.9.7 software [26]. The statistical difference between the two variables was analyzed by unpaired t-test and Pearson's moment correlation to evaluate the relationship between variables. The Chi-square test was used to test the difference in the frequency of VacA or CagA positive in the groups of patients. Graph-pad Prism version 9.0 (San Diego, CA, USA) was used for statistical analysis. A p < 0.05 was considered statistically significant.

## Results

### Clinical and endoscopic findings

The age was not significantly different (p = 0.07) between *H. pylori*-negative and *H. pylori*-positive subjects (Table 1). The age of health check-up subjects (54.0 ± 9.9) and *H. pylori* patients (57.0 ± 15.0) was not significantly different. There was a predominant number of male subjects in both groups. The main reasons for the endoscopic study were symptom complaints (35.1%) and regular health checkups (48.7%) in the *H. pylori*-negative group and symptom complaints (44.0%), and secondary health checkups (32.0%) in the *H. pylori*-positive group. Regarding endoscopic findings, a high percentage (83.8%) of *H. pylori*-negative subjects showed a regular arrangement of collecting venules (RAC) compared to their *H. pylori*-positive counterpart. Extra-gastric manifestations in each group are described in Table 1.

**Table 1:** Clinical profile of the subjects

	*H. pylori*-negative (%)	*H. pylori*-positive (%)
No of subjects	37	25
Sex		
Males	24 (64.9)	14 (56)
Females	13 (35.1)	11 (44)
Age (mean ± SEM)	53.51 ± 2.16	59.64 ± 2.67
Diagnosis of *H. pylori* infection		
Endoscopic findings	37	25
Anti-*H.pylori* antibody	37	25
Histology	2	5
Urea breath test	4	4
Purpose of endoscopic study		
Gastric cancer screening	1 (2.7)	2 (8)
Gastric polypectomy	0 (0)	0 (0)
Symptom complaint	13 (35.1)	11 (44)
No symptoms	18	2
Desire for detailed examination	5	9
Epigastric pain	4	4
Nausea	3	3
Abdominal discomfort	3	0
Chest burning	3	1
Epigastric discomfort	2	3
Anorexia	1	2
Vomiting	0	1
Others	4	2
Regular health checkup	18 (48.7)	3 (12)
Secondary health checkup	5 (13.5)	8 (32)
Extra-gastric manifestations		
Hyperlipidemia	11	7
Fatty liver	6	1
Hyperuricemia	4	1
Diabetes mellitus	3	1
Hypertension	3	3
Liver injury	2	1
Gallbladder polyp	2	0
Gallbladder stone	2	1
Colon polyp	2	0
Arrhythmia	1	2
Others	10	6
Endoscopic findings		
RAC pattern ( ±)	31/6 (83.8/16.2)	3/22 (12/88)
Atrophy (CO-1/C2-3/O1-3)	27/9/1 (73.0/24.3/2.7)	2/17/6 (8/68/24)

A patient can have more than symptoms or complications

Number in () indicate %

*RAC* regular arrangement of collecting venules

Based on the Kimura and Takemoto classification system for gastric mucosal atrophy, a high percentage of *H. pylori*-negative subjects (Co-1, 73%) and *H. pylori*-positive subjects (C-2-3, 68%) showed a closed type of gastric mucosal atrophy (Table 1).

### Increased circulating VacA, CagA, and IL-12p70 during *H. pylori* infection

In an attempt to demonstrate the presence of VacA and CagA in the systemic circulation of subjects with *H. pylori* infection, we developed immunoassay systems using commercially available anti-VacA and Anti-CagA antibodies. Based on our criteria to define the presence or absence of VacA or CagA, 5 (20%) out of 25 *H. pylori*-positive patients were positive for serum levels of VacA (p =  0.02) and CagA (p = 0.05). However, using the same criteria, among 37 *H. pylori*-negative patients 1 was positive for serum VacA and 2 patients for serum CagA. The serum concentration of IL-12p70 was also significantly higher in *H. pylori*-positive subjects than in the *H. pylori*-negative group (Fig. 1b, c). These results suggest the presence of VacA and CagA antigens in the systemic circulation of some patients with *H. pylori* infection.

### Correlation of VacA and CagA with anti-*H. pylori* antibody and IL-12p70

**Fig. 2:**
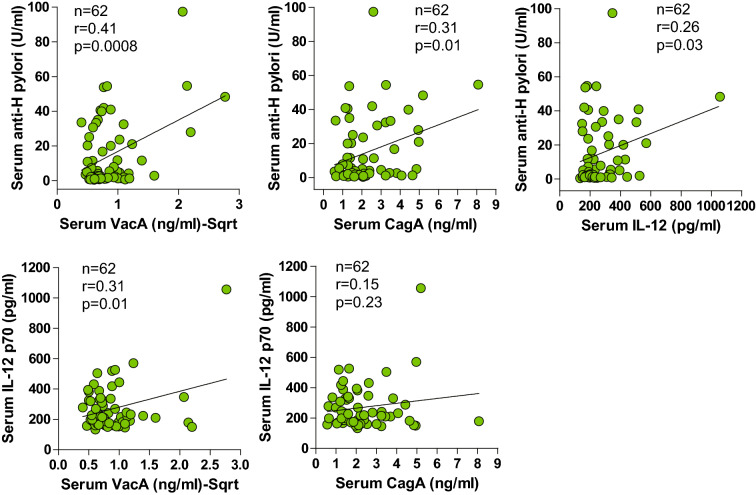
Correlation of vacuolating cytotoxin A, and cytotoxin-associated gene A with several parameters. Anti-*Helicobacter pylori* antibody, vacuolating cytotoxin A (VacA), cytotoxin-associated gene A (CagA) protein and interleukin-12p70 (IL-12) were measured using immunoassays. Data are expressed as the mean ± SEM. Statistical analysis by Pearson's product moment correlation

We then reasoned that if there are circulating antigens derived from *H. pylori*, they would be correlated with the circulating level of anti-*H. pylori* antibody and the inflammatory cytokine IL-12p70. Data from both *H. pylori*-negative and *H. pylori*-positive patients were included in the correlation analysis. The results demonstrated a significant correlation of serum VacA and CagA with the serum concentrations of anti-*H. pylori* antibody and IL-12p70 in all subjects. As expected, the serum level of anti-*H. pylori* antibody was significantly correlated with IL-12p70 in all subjects (Fig. 2). These results further support the possible spill-over of VacA and CagA antigens in the systemic circulation of patients with *H. pylori* infection.

## Discussion

This study suggests that some patients with *H. pylori* infection have circulating levels of VacA and CagA that correlate with anti-*H. pylori* antibody and IL-12p70.

The role of *H. pylori* infection in the pathogenesis of gastric diseases, including chronic gastritis, peptic ulcer, MALT lymphoma, and gastric adenocarcinoma, is irrefutable [1, 2, 27]. Virulence factors including VacA and CagA play crucial roles in the pathogenic activity of the bacterium. VacA induces autophagy, autophagosomes, or the formation of intracellular vacuoles in host epithelial cells enabling colonization and survival of *H. pylori* in the gastric mucosa [28, 29]. CagA is a toxin that, together with a type 4 secretion system or T4SS, is encoded by the *cag* pathogenicity island that plays a central role in *H. pylori*-associated gastric cancer [28]. However, contrary to the convincing evidence on the role of VacA and CagA in gastric diseases, their pathological implication in the *H. pylori* infection-associated extragastric manifestations remains unexplored.

Extragastric manifestations are diseases that occur in association with *H. pylori* infection [30]. Among many others, they include hematological disorders (primary immune thrombocytopenia), skin diseases (rosacea, chronic urticaria, alopecia areata), inflammatory bowel diseases (chronic ulcerative colitis, Crohn's disease), metabolic disorders (diabetes mellitus, metabolic syndrome), neurological diseases (Alzheimer's disease, Parkinson's disease, Guillain-Barrė syndrome) and autoimmune diseases (rheumatoid arthritis, systemic lupus erythematosus) [30–33]. Several mechanisms have been reported to mediate these extragastric distant effects of *H. pylori* infection, including induction of autoantibodies due to molecular mimicry between host proteins and *H. pylori*-derived antigens and excessive release of inflammatory mediators (e.g., cytokines, nitric oxide, eosinophilic cationic protein) [32, 34–36].

Direct involvement of *H. pylori*-derived virulence factors including VacA and CagA in the pathogenesis of extragastric disorders is unknown. If there is a direct effect, it would imply the occurrence of spillover of VacA and CagA antigens into the systemic circulation. To demonstrate this hypothesis, we developed immunoassay systems for VacA and CagA to measure their circulating levels in subjects with *H. pylori* infection. The results of our present study suggest that some patients with *H. pylori* infection have circulating levels of VacA and CagA. In addition, the serum level of anti-*H. pylori* antibody significantly correlated with circulating VacA and CagA in all patients. These observations suggest that patients with *H. pylori* infection may have spill-over of VacA and CagA antigens in the systemic circulation. A report showing that serum exosomes from patients with *H. pylori* infection contain CagA and that exosomes containing CagA are expressed by gastric epithelial cells supports our present findings [37]. Furthermore, a systemic inflammatory response with an increased circulating level of IL-12 is also a characteristic finding during *H. pylori* infection [38, 39]. In agreement with this, we found that a high circulating level of IL-12p70 significantly correlates with anti-*H. pylori* antibody and VacA. It is worth noting here that some of our *H. pylori*-negative patients showed serum levels of VacA and CagA that were higher than the upper cutoff used to judge the presence or absence of circulating antigens, suggesting the need to improve the sensitivity and specificity of the immunoassays for serum VacA and CagA.

The small population size, the use of polyclonal antibodies for the measurement of VacA and CagA, the low sensitivity and specificity of the immunoassays, and the fact that the study was conducted in a single institution are limitations of the present investigation.

## Conclusion

This study suggests that spill-over of VacA and CagA antigens in the systemic circulation may occur in patients with *H. pylori* infection. However, further studies should be carried out to corroborate these findings.

## Data Availability

All data generated or analyzed during the current study are included in the article. Also, any data and materials are available from the corresponding authors upon reasonable request.
